# Practical Notes on Diagnosis and Treatment

**Published:** 1910-10-08

**Authors:** 


### PRACTICAL NOTES ON DIAGNOSIS AND TREATMENT.

**Obstruction.**

Complete intestinal obstruction, if not accompanied by strangulation, and especially if there has been previous incomplete obstruction, may persist for several days without producing any marked quickening of the pulse.—*Mr. Sampson Handley.*

**A Meat Diet.**

The digestibility of finely divided meat is not sufficiently appreciated; probably no food is tolerated so well by our stomachs, for even where the gastric chemistry is deficient such food is easily got rid of and causes no discomfort.—*Dr. Saundby.*

**Diagnosis of Cancer of the Liver.**

If an enlargement of the liver is accompanied by one only of the symptoms of jaundice and ascites, then we may make the important generalisation that the case is not one of cancer of the liver. Jaundice is no part of carcinoma of the liver; when it occurs it is an accident, due to pressure of enlarged glands on the portal fissure, and then these would be large enough to cause ascites by pressing on the larger and softer portal vein.—*Dr. Sidney Phillips.*

**Ocular Diseases and the Nasal Passages.**

The researches demonstrating so fully the intimate relations between the accessory nasal sinuses and the orbits and the optic canals have naturally attracted attention to the possible and probable ophthalmic complications and sequelæ of nasal disease. A careful examination of the nasal passages is now indicated in all instances of obscure and persistent headache and neuralgia, where refraction error and muscular unbalance have been eliminated; also, among other affections, in cases of slight orbital œdema, cellulitis, and thrombosis of the cavernous sinus without an evident aural cause.—*Mr. Vernon Cargill.*

**Fæcal Vomiting.**

Fæcal vomiting is not to be regarded as a symptom of intestinal obstruction but as a herald of approaching death.—*Mr. A. Baldwin.*

**Effects of Copaiba.**

The symptoms of acute intestinal obstruction with violent pains and vomiting have been observed in patients who were taking copaiba in the course of treatment for gonorrhœa.—*Mr. Lockwood.*

**Urinary Septicæmia.**

The tongue is an important index of the state of the renal function. Thirst and a dry tongue are frequent symptoms of interference with the action of the kidneys. Headache is another frequent feature of these cases. In the later stages of "urinary septicæmia" the face has a peculiar yellow earthy look, that is almost characteristic. Hiccough is a sign of grave import, and its ocurrence after operation on the lower urinary tract may be looked up as a signal for alarm.

**Value of a Proteid Diet.**

The details of preparation and regulation of the diet are of paramount importance if a successful issue is to be looked for. The basis of the exclusive proteid diet consists of cooked minced lean beef, so prepared that all gristle and most of the connective tissue are removed. From two ounces upwards of the meat are to be given at five-hourly intervals three times a day. Hot water is also an essential part of the diet, and is given four times a day, more than an hour before each meal and about three after the meal. The water should be sipped slowly at a temperature of 120° F. Speaking generally, the diet should be reserved for chronic disorders, such as chronic hyperchlorhydria, dilatation of the stomach, etc.—*Dr. Ernest Young.*

